# Dengue virus infection in children: Serum lipidomics profiling for biomarker discovery

**DOI:** 10.1371/journal.pntd.0013691

**Published:** 2025-11-24

**Authors:** Ricardo E. Correa Fierro, Noroska Gabriela Mogollón Salazar, Washington B. Cárdenas, Evencio Joel Medina-Villamizar, Jefferson Pastuña-Fasso, Melanie Ochoa-Ocampo, Giovanna Morán-Marcillo, Mary Ernestina Regato Arrata, Mildred Zambrano, Joyce Andrade, Juan Chang, Saurabh Mehta, Fernanda Bertuccez Cordeiro

**Affiliations:** 1 Laboratorio para Ensayos Biológicos, Biológicos y Diagnósticos-ENBIDIA, Facultad de Ciencias de la Vida, ESPOL, Guayaquil, Ecuador; 2 Biomolecules Discovery Group, Universidad Regional Amazónica Ikiam, Tena, Napo, Ecuador; 3 School of Industrial, Computer and Aeronautical Engineering, University of León, León, Spain; 4 Instituto Nacional de Investigación en Salud Pública INSPI, Guayaquil, Guayas, Ecuador; 5 Servicio de Infectología e Epidemiología, Hospital de Niños Dr. Roberto Gilbert, Guayaquil, Guayas, Ecuador; 6 Cornell Joan Klein Jacobs Center for Precision Nutrition and Health, Cornell University, Ithaca, New York, United States of America; Laboratorio Arbovirus Instituto Virologia, ARGENTINA

## Abstract

Dengue fever is a significant global health concern, particularly in tropical and subtropical regions, where it disproportionately affects children and adolescents. The disease, caused by the dengue virus (DENV), triggers a complex immune response that leads to metabolic alterations, particularly in lipid metabolism, which plays a key role in inflammation and disease progression. Despite advancements in diagnostic methods, the search for novel biomarkers may support the development of new diagnostic tools for faster patient screening. In this study, we applied a lipidomics approach using liquid chromatography-mass spectrometry to analyze the serum lipid metabolome of children and adolescents infected with DENV (n = 25) compared to controls (n = 15). Multivariate statistics included partial least squares discriminant analysis (PLS-DA) to assess group separation and receiver operating characteristic (ROC) curve analysis to evaluate biomarker performance. The PLS-DA revealed a tendency of separation between groups, with component 5 showing the highest predictive power (Q2 = 0.68345). From this data, 12 metabolites were significantly more abundant in controls, while 3 were more abundant in DENV infected group. ROC curve analysis demonstrated a sensitivity of 80% for the metabolite of *m/z* 246.265, and a sensitivity of 96% for all metabolites, as a set. The metabolites were attributed to sphingolipids, fatty acids, glycerol lipids, and sterols. Our findings reveal significant lipid metabolic alterations in pediatric dengue fever, highlighting their biomarker potential. This study reinforces the value of lipidomics in dengue research and biomarker discovery, which may contribute to the development of diagnosis tools that will improve patient care.

## Author summary

Dengue fever is a mosquito-borne viral disease that affects millions of people worldwide, with higher incidence in tropical and subtropical regions. Children and adolescents are particularly vulnerable, which makes early detection a crucial step for effective management. In our study, we explored how dengue infection alters lipid metabolism in the blood, as lipids play a key role in the immune response and inflammatory processes. Using an advanced technique called lipidomics, we identified specific molecules that differ between children with dengue and non-dengue febrile controls. Our findings suggest that the lipids found could serve as potential biomarkers to help distinguish dengue cases from non-dengue febrile controls. This could contribute to the development of better diagnostic tools, generating screening tests for faster detection of arboviruses and therefore improving patient care. However, further research with larger groups of patients is needed to confirm these results and explore their clinical applications. By understanding how dengue affects metabolism, we move closer to improving diagnostics and patient outcomes.

## Introduction

Dengue fever (DF) is considered a significant global health concern by the World Health Organization (WHO) [1]. DF is caused by an arthropod-transmitted virus, involving mosquitoes of the genus *Aedes* as a natural host vector [2]. In 2023, an unexpected surge of DF resulted in over five million cases and more than 5,000 deaths in the most affected regions [3]. Of these cases, almost 80% have been reported in countries where DF is endemic [3]. Nevertheless, these numbers may be underestimated due to variability in symptoms and lack of epidemiological surveillance for arbovirus infections [4].

DF can progress to life-threatening clinical features such as bleeding, organ involvement and plasma leakage leading to respiratory distress and shock, currently defined as severe dengue fever (SDF) [5]. Particularly in children, the clinical presentation of DF often includes high-grade fever, petechial rashes, abdominal pain, severe headache, and hepatomegaly whereas edema of the lower extremities, vomiting, retro-orbital puffiness, and seizures are common clinical signs and symptoms [6,7]. The acute phase of DF requires a molecular diagnosis, which involves detecting the viral genetic material in patient samples by using a real-time reverse transcription polymerase chain reaction approach (qRT-PCR) [8,9]. The qRT-PCR is highly specific and sensitive during the early stages of infection (usually within the first 5–7 days after symptom onset), when viral loads are high [10]. This method allows for the detection and even serotyping of dengue virus, which is critical for early clinical decision-making and epidemiological surveillance. However, its sensitivity significantly decreases in the later stages of infection as viremia declines. In such cases, serological assays that detect anti-DENV IgM and IgG antibodies become more appropriate [11]. These assays are useful for identifying recent (IgM) or past (IgG) infections and are widely accessible and relatively inexpensive. Nonetheless, they have limitations, including cross-reactivity with other viruses, such as Zika, delayed antibody response that may lead to false negatives if performed too early, and the potential for persistent IgM detection weeks after infection, complicating the interpretation of acute versus past infection [11].

In addition to the changes in immunoglobulin levels throughout the course of infection, DF may cause alterations to the abundance of various inflammatory lipids and metabolites [5]. To analyze these compounds, metabolomics has emerged as an attractive tool to identify changes in metabolic processes within a biological system after a stress event, such as arboviral infections [12].

The development of metabolomics-based mass spectrometry presents great potential for diagnosis [13,14]. Additionally, ultra-efficient liquid chromatography-mass spectrometry (LC-MS) has contributed to the understanding of several disease mechanisms besides driving the development of diagnostic tools [15]. For DF, LC-MS has been used in the discovery of molecules associated with viral pathogenicity, although few studies focused on patients’ health [16,17]. LC-MS has many analytical advantages, such as the pre-separation of sample components by liquid chromatography, reducing the complexity of the sample before the detection of metabolites by mass spectrometry [18,19].

Therefore, this study focuses on the serum lipidomic profile of children with DF. We hypothesize that dengue virus (DENV) infection in children induces specific alterations in the serum lipidomic profile, which can be identified through untargeted LC–MS metabolomics and may propose novel biomarkers for the disease. The untargeted metabolomics in the present study enabled the identification of endogenous compounds potentially linked to metabolic dysregulation in DENV infection. These potential biomarkers may improve the comprehension of metabolic pathways involved in dengue pathogenesis in pediatric patients and may be used in diagnosis in the future.

## Materials and methods

### Ethics statement

**Fig 1:**
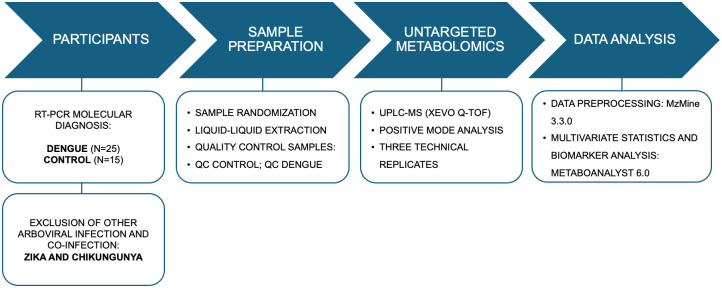
Workflow of untargeted metabolomics in serum samples from children infected with DENV.

This study was conducted in collaboration with the “Dr. Roberto Gilbert Children Hospital” in Guayaquil, Ecuador. The study was approved by the Research Ethics Committee of the Luis Vernaza Hospital under protocol number HLV-DOF-CEI-004, evaluated and approved in February 2017. Given the inclusion of pediatric patients, written informed consent was obtained from the legal guardians of all children prior to sample collection. Blood samples were collected only from children who presented symptoms consistent with arboviral infection and required clinical evaluation for diagnostic purposes. All procedures performed in this study were in accordance with ethical standards from the 1964 Helsinki declaration and its later amendments or comparable ethical standards. The study workflow is represented in Fig 1.

### Participants and samples

Patients under 18 years of age were included in this study if they presented at least one of the signs or symptoms of arboviral infection within the first five days of clinical presentation, due to its association with the viraemia period when the virus can be detected in serum, plasma and whole blood from 0 to 7 days following the beginning of symptoms [10]. These signs and symptoms comprised fever; headache; retro-orbital pain; myalgia; arthralgia; nausea; vomiting; or rash. DF diagnosis was confirmed by reverse-transcription polymerase chain reaction (RT-PCR, N = 25). The control group was composed of patients under 18 years old that attended the. Hospital due to similar febrile symptoms. These patients’ samples were also tested by RT-PCR to detect arboviral infection and were negative for DENV, Zika and Chikungunya (N = 15). Participants exhibiting signs and symptoms characteristic of the following conditions were excluded from the study: otitis media, sinusitis, urinary tract infection and allergic reactions. In addition, RT-PCR was also carried out to identify other arboviral infections and co-infections, such as Zika and Chikungunya; samples that tested positive for these viruses were excluded. Individuals with clinical conditions known to significantly affect their metabolism were also not included in this study.

Blood samples from all patients were collected. A single puncture was performed, and a vacutainer system was used to collect approximately 4 mL in sterile tubes without anticoagulant to obtain serum used for molecular diagnosis and metabolites extraction.

### Molecular diagnosis of dengue

For molecular diagnosis by RT-PCR, 250 µL of serum was homogenized with 750 µL of TRI Reagent (Sigma-Aldrich Inc. MO, USA) and incubated for 10 minutes to allow lysis. Chloroform was added to enhance the separation of aqueous and organic compounds. After mixing, a phase separation was performed by centrifugation at 14000 *× *g for 15 minutes at 4 °C. RNA suspended in the upper layer was transferred to a fresh tube containing glycogen and sodium acetate for a better pellet visualization. The pellet containing RNA was then precipitated with ice-cold 2-propanol and centrifuged for two consecutive wash steps with ethanol (75%), completely dried and resuspended in 25 µL of TE buffer or UltraPure water until molecular amplification.

### Reverse-transcription polymerase chain reaction

Dengue mono-infection was confirmed by reverse-transcription polymerase chain reaction (RT-PCR) using the SuperScript III Platinum One Step RT-PCR Kit (Invitrogen; CA). A two-step heminested protocol used a sense primer: 5’-TCAATATGCTGAAACGCGCGAGAAACC-3’ and anti-sense primer: 5’-TTGCACCAACAATCAATGTC-3’, targeting the C-prM region of the POLY gene, for all 4 serotypes (DENV1, DENV2, DENV3 and DENV4) molecular diagnosis. Additionally, RT-PCR was also performed in plasma and whole blood to assure that participants were infected with dengue and discard co-infections, such as Zika and Chikungunya.

### Sample preparation for metabolomics

For metabolomics, serum samples were obtained after centrifugation at 2000 *× g* for 10 minutes at 4 °C, distributed in aliquots of 1.5 mL and maintained at -80 °C until metabolites extraction. Metabolite extraction was performed after the collection of all samples to minimize variability and avoid bias during the extraction. Additionally, samples from both groups were randomized prior to metabolites extraction to prevent data analysis from potential issues such as LC-MS signal drift, batch effects, and operator-related variability, ensuring reliable and reproducible results.

Metabolite extraction was carried out by adding 1 mL of solvent mixture in a (1:3:1) ratio of Chloroform/Methanol/Water to 25 µL of serum [20]. Chloroform (≥99.9%) from Sigma-Aldrich (Saint Louis, MO, USA), HPLC grade Methanol hypergrade for LC-MS purchased from Merck KGaA (Darmstadt, Germany) and UltraPure DNase/RNase-Free Distilled Water from Thermo Fisher Scientific Inc (Waltham, MA, USA) were used to prepare the solvent mixture in sterile glass. The samples were vortexed for 30 seconds and then centrifuged at 13000 × g for 3 minutes. This extraction protocol was carried out at 4 °C and extraction conditions were identical in all samples. Two pooled samples were prepared from 5 μL collected from each extract. The pools of metabolites were used as quality control samples (QC) for dengue (QCD) and for controls (QCC) groups, without the use of internal standards for both samples and QC. The amount of 200 μL of supernatant was transferred to a new microtube, and metabolites were concentrated in a vacuum concentrator (miVac Duo Concentrator, GeneVac, UK). Dried samples were then resuspended in 50 μL of UltraPure water with formic acid 0.1% (Merck KGaA, Darmstadt, Germany) and transferred to glass vials for analysis.

### LC-MS untargeted metabolomics

Metabolomics was carried out using a Waters Model I-Class ultra-performance liquid chromatography (UPLC) coupled with a Xevo G2QTOF mass spectrometer (Waters Corporation, MA, USA). The liquid chromatography was performed on an ACQUITY UPLC BEH C18 column (Waters Corporation, MA, USA) measuring 1.7 μm in particle size, with dimensions of 100 mm × 2.1 mm i.d. The flow rate was set at 0.5 mL/min. The mobile phases consisted of water with formic acid 0.1% (phase A) and acetonitrile with formic acid 0.1% (phase B). A total of 10 μL of the sample was employed for the analysis, and the elution gradient was established as follows: 80% A and 20% B for 1 minute, followed by a linear transition to 50% A and 50% B for the next minute. Subsequently, the gradient was adjusted to 35% A and 65% B for 2 minutes. Next, 20% A and 80% B for 3 minutes. The gradient was further modified to 3% A and 97% B for the next 10 minutes. Finally, the conditions were restored for column washing and conditioning for 4 minutes. The total run time per sample was 21 minutes. Each sample was run in three technical replicates.

For mass spectrometry, electrospray ionization (ESI) source was used in positive mode, and data were acquired in full scan. The capillary voltage was set at 0.5 kV, with a cone gas flow rate of 30 L/h and a desolvation gas flow rate of 900 L/h. The source temperature was 120 °C, while the desolvation temperature was 450 °C. Both the sampling cone and source compensation were adjusted to 40 and 80 V, respectively. Scan rate of 1 s and covering a mass range of *m/z* 100–1200 Da was used in full scan mode.

To ensure data quality, MS data was obtained from the QC samples from dengue and control groups. The QC samples were injected into the LC–MS instrument after every 5 samples injection, along the analytical block to condition and equilibrate the system. Data from QC samples were used to graph the retention time drift and as a reference for data processing. To assess the quality of the experiment, Principal Component Analysis (PCA) was performed including QCC and QCD pools showing that QC samples clustered closely with their respective groups (S1 Fig).

The raw data from LC-MS (*.raw) was converted to *.mzML format using ProteoWizard [21] Then, the *.mzML files were processed in MS-DIAL ver. 4.9.221218 (http://prime.psc.riken.jp/) considering peak detection, alignment, gap filling, and blank filtering (maximum sample intensity/average blank intensity ratio > 7) according to parameters shown in S1 Table. The MS-DIAL feature list table *.txt was exported for statistical analysis.

Prior to statistical analysis, we preprocessed the MS-DIAL-exported data to obtain high-quality data [22]. The data preprocessing was implemented in R 4.2.2 using “notame” package (https://github.com/antonvsdata/notame). Preprocessing included drift correction by smoothed cubic spline, batch correction by mean/median difference of QCs, and data imputation by the random forest algorithm.

This study is available at the NIH Common Fund’s National Metabolomics Data Repository (NMDR) website, the Metabolomics Workbench, https://www.metabolomicsworkbench.org where it has been assigned Study ID ST003780.

### Statistical analysis

After LC-MS data preprocessing, the *.csv file was submitted to multivariate statistics in Metaboanalyst 5.0 software (http://www.metaboanalyst.ca/). An autoscaling normalization was performed to adjust each variable by a scaling factor. The statistical analysis was carried out by partial least squares discriminant analysis (PLS-DA), which performs a supervised classification and generates a regression model via linear combination of the original variables.

Next, the PLS-DA regression model was submitted to cross-validation test, in which a prediction index Q2 was established to determine the PLS-DA prediction ability [23]. The R2 (coefficient of determination) value was obtained to assess how well the model fits the data. For the cross-validation, 5 components were included in the search and the chosen method was 5-fold. Additionally, metabolites of relevance were determined by the variables of importance for the projection (VIP) scores of the regression model, exhibiting significant features recognized by PLS-DA, as well as the relative concentrations of the corresponding metabolites within each group.

### Compound identification

Lipids attribution was performed based on a multi-level characterization of the 15 putative *m/z* suggested in the panel of metabolites with better variable importance in projection (VIP scores > 1.9) from the component with the best Q2 performance measure of the PLS-DA model. The attribution was performed at LipidMaps database (https://www.lipidmaps.org/), in Bulk structure search with a tolerance of +/- 0.05 *m/z* on the positive mode. We included all possibilities of metabolites from the following main classes: Fatty Acids; Glycerolipids; Glycerophospholipids; Sphingolipids; and Sterol Lipids. Although other databases were used, such as Human metabolome Database (HMDB) and Metabolomics Workbench, data found were related to metabolites that were not endogenous and therefore were not included as main results (S3 Table). Lipid names follow the LIPID MAPS shorthand nomenclature. The number before the colon indicates the number of carbon atoms, and the number after the colon indicates the number of double bonds (e.g., FA 14:0 = 14 carbons, 0 double bonds). A semicolon (;) is used to separate additional structural descriptors. O: hydroxyl groups (e.g., O2 = two hydroxyls). S: defined stereochemistry. GlcA: glucuronic acid moiety, or other functional modifications. For glycerolipids, a comma (,) separates multiple fatty acyl or alkyl chains (e.g., PC(16:0,18:1) if present). In some cases, prefixes such as O- indicate ether-linked chains (e.g., DG O-36:9;O). This notation allows a concise representation of complex lipid structures in alignment with mass spectrometry–based identification.

### Biomarker analysis

The biomarker potential of the lipid metabolites with higher discrimination proposed by PLS-DA was evaluated using Receiver Operating Characteristic (ROC) curve analysis to calculate sensitivity and specificity. The classical univariate ROC curve analysis was applied to each metabolite to calculate their area under the curve (AUC) and their 95% confidence interval. For combined metabolites, the ROC curve-based model was used, in which all metabolites from PLS-DA were evaluated.

## Results

**Fig 2:**
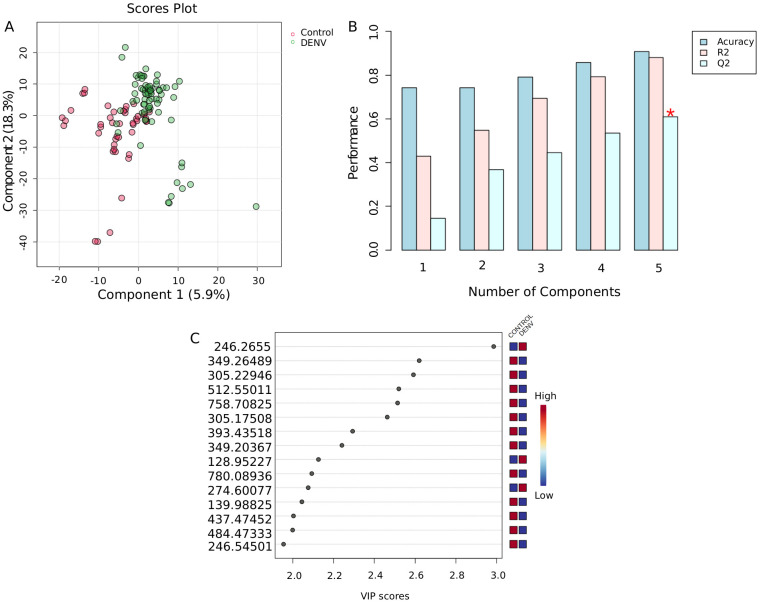
PLS-DA regression model from untargeted metabolomics of serum samples from children infected with dengue and controls. **A.** PLS-DA 2D Score plot exhibiting a tendence of separation between groups. **B.** Cross-validation of PLS-DA shows a Q2 of 0.7 for the regression model. **C.** VIP scores show a metabolite panel with 15 *m/z* with differential abundances between dengue and controls. PLSDA, Partial least square-discriminant analysis. VIP, variable importance in projection.

Our study included a total of 40 individuals from 0 to 18 years old. The average age for both groups was of 6 years old. Untargeted metabolomics of serum samples from children infected with dengue was compared to a control group. Multivariate statistics with PLS-DA showed a tendency of separation between groups (Fig 2A), which indicates that the metabolite profiles from the studied groups show distinct patterns, although the visual separation observed in the chart is not complete. The PLS-DA cross-validation showed that component 5 had the higher predictive ability of the model, represented by Q2 = 0.68345 and R2 = 0.88095 (Fig 2B). The Q2 value is a measure of the predictive ability of the PLS-DA. The value found in our study indicates that 68.3% of the variation in the group classification (dengue vs. control) can be predicted by the model based on the metabolite features. The R2 value indicates that 80% of the variation in the dataset is explained by the PLS-DA model.

Detailed information about cross validation is presented in S2 Table. As a result of the PLS-DA model, the Variable Importance in Projection (VIP) chart showed that 12 metabolites were of higher abundance in controls, whereas 3 metabolites were of higher abundance in dengue samples (Fig 2C). The VIP scores are higher than 2, demonstrating the metabolites that are significant for the discrimination between groups.

Compounds attribution was carried out in LIPID MAPS structure database applying a mass tolerance of +/- 0.05 *m/z* presented in Table 1. The metabolite search showed initially that 239 metabolites could be possible for the *m/z* uploaded. From 15 metabolites, 5 were not attributed to a main class. Other metabolites were attributed to more than one main class, considering their delta variation. The main classes found were sphingolipids, fatty acids, glycerol lipids and sterol. The complete table of attribution is shown in S3 Table.

**Table 1:** Lipids attribution for controls and dengue groups according with Lipid Maps database, obtained via exploratory metabolomics from human serum.

Group	Mass	Delta	Main Class	Name	Formula	Adduct
Dengue	246.2655	0.0228	Sphingolipid	SPB 14:0;O2	C14H31NO2	[M+H]+
			Fatty Acid	FA 14:0	C14H28O2	[M+NH4]+
Control	349.26489	0.0027	Sphingolipid	SM 34:4;O2	C39H73N2O6P	[M+2H]2+
Control	305.22946	0.0002	Di(acyl|alkyl)glycerol	DG 36:8	C39H60O5	[M+2H]2+
			Di(acyl|alkyl)glycerol	DG O-36:9;O	C39H60O5	[M+2H]2+
			Tri(acyl|alkyl)glycerol	TG O-36:8	C39H60O5	[M+2H]2+
Control	512.55011	0.0464	Sphingolipid	Cer 32:0;O2	C32H65NO3	[M+H]+
			Fatty Acid	NAE 30:0;O	C32H65NO3	[M+H]+
			Fatty Acid	FA 32:1;O	C32H62O3	[M+NH4]+
Control	758.70825	0.0042	Tri(acyl|alkyl)glycerol	TG 100:10	C103H182O6	[M+2H]2+
			Tri(acyl|alkyl)glycerol	TG O-100:11;O	C103H182O6	[M+2H]2+
Control	305.17508	0.0004	Sterol Lipids	ST 18:3;O4	C18H24O4	[M+H]+
			Fatty Acid	FA 18:5;O3	C18H26O5	[M+H-H2O]+
			Sterol Lipid	ST 18:2;O5	C18H26O5	[M+H-H2O]+
			Sterol Lipid	ST 30:7;O2;GlcA	C36H48O8	[M+2H]2+
Control	393.43518	–	Not Attributed	–	–	–
Control	349.20367	0.0017	Sterol Lipid	ST 18:0;O;S	C18H30O4SLi	[M+Li]+
Dengue	128.95227	–	Not Attributed	–	–	–
Control	780.08936	–	Not Attributed	–	–	–
Dengue	274.60077	0.0027	Glycerophospholipid	LPT 16:4	C23H38NO10PNa2	[M+2Na]2+
Control	139.98825	–	Not Attributted	–	–	–
Control	437.47452	0.0392	Mono(acyl|alkyl)glycerol	MG O-26:1	C29H58O3	[M+H-H2O]+
Control	484.47333	0.0009	Sphingolipid	Cer 30:0;O2	C30H61NO3	[M+H]+
			Fatty Acid	NAE 28:0;O	C30H61NO3	[M+H]+
			Fatty Acid	FA 30:1;O	C30H58O3	[M+NH4]+
Control	246.54501	–	Not Attributted	–	–	–

*Not Attributed refers to m/z that was detected a potential biomarker by PLS-DA, although main class was not identified by LipidMaps.

**Fig 3:**
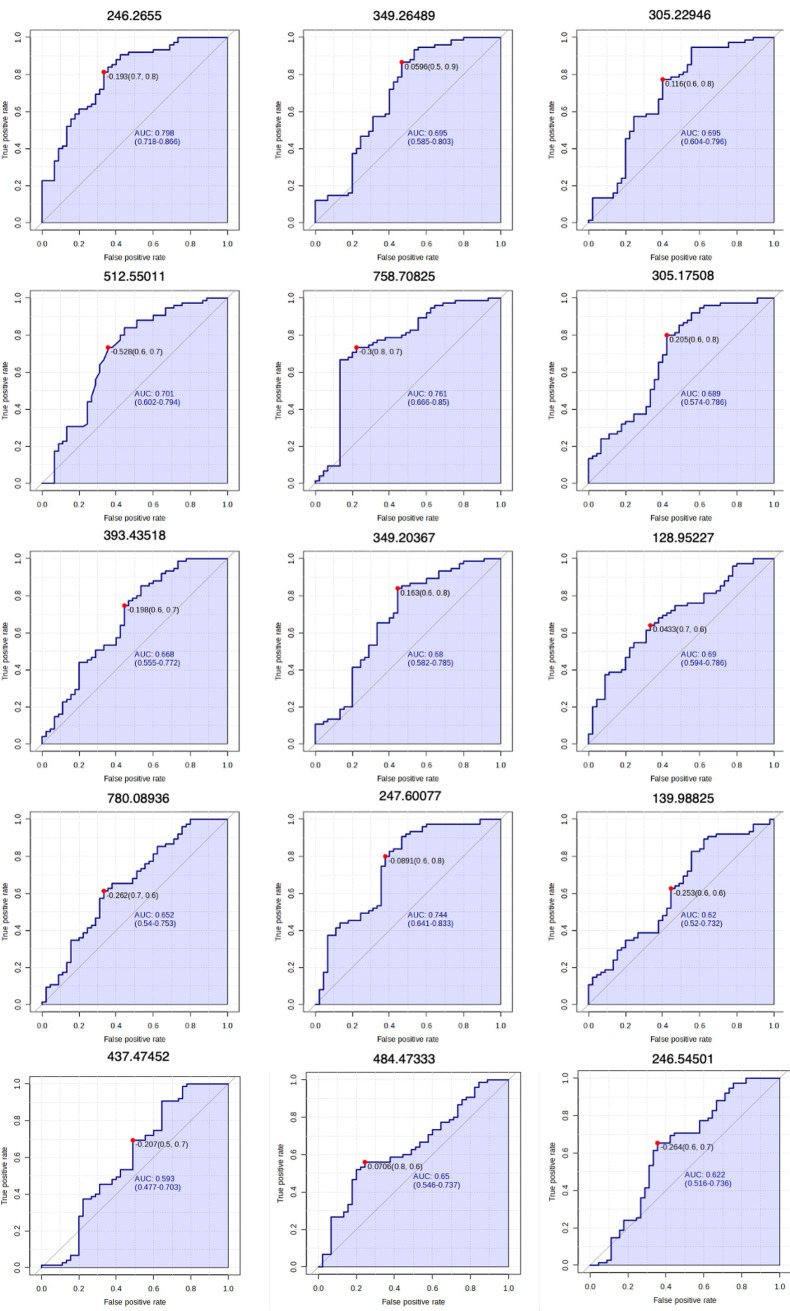
Univariate ROC curves for individual analysis of the 15 *m/z* suggested as biomarkers. The horizontal coordinates indicate the false positive rate; the vertical coordinates indicate the true positive rate; and the area under the curve (AUC) value indicates the prediction accuracy. ROC, Receptor operating characteristics.

**Fig 4:**
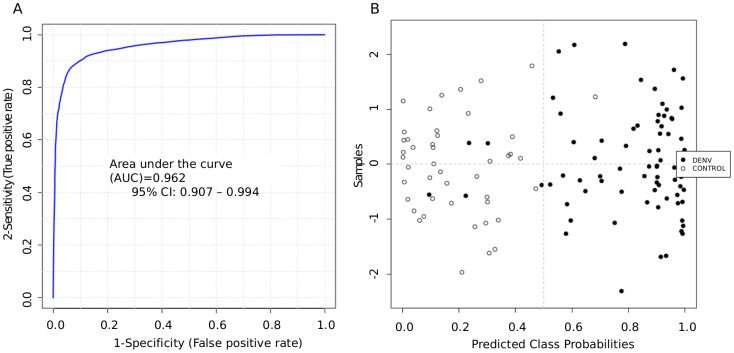
ROC curve analysis of a set of biomarkers obtained via untargeted metabolomics in serum samples from children infected with DENV compared to Control. **A.** ROC curve view shows an area under ROC curve (AUC) of 0.962. **B.** Tester analysis shows 93% (70 out of 75) of correct classification. ROC, Receptor operating characteristics.

Considering the biomarker analysis by ROC curve, individual metabolites analysis showed that *m/z* 246.2655 presented the highest sensitivity (AUC = 0.798, 95% CI: 0.718-0.866), while the *m/z* 437.47452 presented the lowest sensitivity (AUC = 0.59, 95% CI: 0.48-0.703). All individual metabolites’ ROC curves are shown in Fig 3. For the combined metabolites, the ROC curve model evaluation showed a high sensitivity (AUC = 0.962, 95% CI: 0.907-0.994 – Fig 4A). The tester showed that from 75 replicates of dengue samples, 70 were properly classified at the dengue group. From controls, 44 out of 45 samples were correctly classified (Fig 4B).

## Discussion

Although dengue is endemic across South America, Central America, and the Caribbean, the region experienced an unprecedented surge in dengue cases starting in early 2023. From Epidemiological Week 1–49 of 2023, the Americas reported over 4.19 million cases, including nearly 4,800 severe cases and approximately 1,747 deaths, marking the highest annual total recorded in the South American subregions [24]. In the WHO Region of the Americas, dengue cases exceeded those of any previous year—with more than 4.1 million suspected cases, including 6,710 severe cases and 2,049 deaths by December 2023 [25]. Given the significant impact of dengue on vulnerable age groups, it is crucial to recognize that children below 14 years old and adults older than 50 are at higher risk of severe cases, including increased rates of hospitalization and mortality [26].

Currently, there is no specific treatment for dengue, although the early diagnosis and identification of signs for severe dengue are crucial to prevent the progression of the disease [3]. Understanding the variability of symptoms and signs in children may assist the differentiation of dengue from other febrile outbreaks [27,28]. DF leads to relevant metabolic alterations that are related to the disruption in the cell’s metabolism and different immunological pathways that are activated [29]. In this context, the application of metabolomics in the study of dengue can detect subtle differences and may be crucial for discovering biomarkers to improve DF diagnosis [29].

In our study, untargeted metabolomics was applied to serum samples from children and adolescents with dengue in comparison with controls. This approach aims to identify as many lipids as possible in different biological matrices [30]. Although LC-MS is considered the gold-standard technology for biomarker discovery, previous metabolomics studies on DF using human samples vary according to the context: the studies show differences in patients’ gender and age; biological matrices used for metabolomics; and different MS approaches, including Gas Chromatography-Mass Spectrometry and proton nuclear magnetic resonance (1H NMR) spectroscopy [31,32].

Multivariate statistics was applied by using PLS-DA, which is one of the most used classification methods in metabolomics [33,34]. In this study, PLS-DA demonstrated a tendency toward separation between the two groups. This method was chosen among other classification models to assure more robustness of the model and interpretability. To support PLS-DA in this study, Q2 and R2 values from cross-validation suggests a moderately strong predictive model, indicating that the studied groups have distinct metabolic profiles and an acceptable model performance without overfitting.

Considering that the metabolites extraction used prioritizes lipids, the present work identified mainly fatty acids (FA), sterol lipids (ST) and glycerolipids (Table 1 and S3 Table). A previous characterization of the serum metabolome of patients with different DF outcomes found a decrease in fatty acid metabolism in DF patients in comparison with SDF life-threatening patients *(known as dengue hemorrhagic fever (DHF) and dengue shock syndrome (DSS)* [35]. Most molecules were polyunsaturated FA (PUFA) that were associated with the severity of dengue [35]. Long-chain polyunsaturated fatty acids, such as docosahexaenoic acid (DHA [FA22:6]) are anti-inflammatory agents that may decrease the production of cytokines and reactive oxygen species [36]. These molecules may inhibit the metabolism of arachidonic acid (AA), one of the main precursors of inflammation [37]. The increase in DHA levels in DF in comparison with SDF might represent a mitigation of immunopathology of DF [35]. In our study, although none of the patients developed SDF, we found a higher abundance of long-chain FA in controls (NAE30:0, FA32:1;0, FA18:5;03, NAE28:0;0, FA30:1;0), which indicates that these molecules may also contribute to mechanism of protection.

However, an analysis of serum metabolome from adults with primary DF revealed increased levels of FA, especially AA [5]. The AAs were attributed to perturbations in fatty acid biosynthesis and β-oxidation [38]. Although AA was not found in our study, the β-oxidation pathway should be further investigated as a potential mechanism of inflammation in DF.

Sterol lipids were more abundant in controls compared to serum from dengue patients. Among these metabolites, cholesterol is one of the most well-studied due to its structural role in cellular membranes and its involvement in DENV pathogenesis [39]. Previous studies show that DENV can manipulate host cholesterol metabolism to facilitate its life cycle. For instance, DENV infection induces the expression of PCSK9, a regulator of cholesterol homeostasis, which may modulate antiviral responses and influence disease severity [39]. Additionally, inhibition of cholesterol biosynthesis has been demonstrated to impair DENV replication, highlighting the virus’s dependence on cholesterol-rich membranes for efficient replication [40]. In our study, the observed reduction in cholesterol levels in dengue patients may reflect the virus’s exploitation of host cholesterol to support its replication process.

Additionally, *in vitro* studies have shown that DENV stimulates *de novo* cholesterol synthesis in the endoplasmic reticulum. Therefore, the cholesterol produced assists in restructuring the endoplasmic reticulum, enabling the formation of replication complexes for DENV [41,42]. Although a potential treatment for dengue would consist of inhibiting *de novo* cholesterol synthesis, clinical trials have not shown enough benefits for the patients [43].

Other metabolites, such as sphingolipids and glycerolipids, were found in both dengue and control groups, but the specific types and abundances varied between them. For sphingolipids, the dengue group showed a higher abundance of sphingolipid bases (SPB 14:0;O2), whereas the control group had higher abundance of ceramides (Cer 30:0;O2). Ceramides are involved in immune modulation and have been shown to activate natural killer T cells, contributing to the antiviral response against DENV [44]. Interestingly, our study found higher ceramide levels in the control group. It is noteworthy that control participants also presented fever but tested negative for arbovirus infection (dengue, zika and chikungunya), suggesting that elevated ceramides may be associated with immune responses, potentially linked to inflammatory pathways rather than being specific to dengue infection.

Controls had higher abundance of di(acyl|alkyl)glycerol (DG), tri(acyl|alkyl)glycerol (TG) and mono(acyl|alkyl)glycerol (MG). These lipids may be associated with oxidative stress during DENV infection and have already been proposed as biomarkers [45,46]. In this context, the oxidative stress caused by dengue generates an overall decrease of triacylglycerol in serum, since TG lipids are highly susceptible to the effect of reactive oxygen species (ROS) [47]. Interestingly, high triglyceride levels have been described as protective molecules against DHF, suggesting it can be used as a predictive biomarker of disease severity. In accordance with previous studies, our study observed lower TG levels in serum from the dengue group, supporting the hypothesis that oxidative stress contributes to TG depletion during infection. While the roles of DG and MG species remain unclear in dengue infection, the literature suggests that these metabolites are more frequently studied in the context of vector biology, particularly in mosquitoes where they contribute to lipid metabolism and virus-host interactions [48,49]. Further research is needed to determine whether alterations in DG and MG levels in human serum reflect similar metabolic pathways or represent novel host responses to DENV infection.

For the dengue group, the glycerophosphothreonines (LPT) were more abundant. The LPT have not been described in either studies of dengue or inflammatory response. However, a similar compound has been related to a modulator-like activity in glucocorticoid receptors [50]. With respect to this activity, the modulation of these receptors may be part of an anti-inflammatory response to prevent exacerbated immune response that may cause SDF [51].

Despite recent interest and frequent use of metabolomics in translational biomedical research, metabolomics-based clinical tests remain unavailable for many diseases due to three main reasons: subtle changes in multiple metabolites; lack of precise metabolite quantification; and inconsistency in study design and reporting, with limited use of standard evaluation methods like ROC curves [52,53]. However, different from traditional methods such as qRT-PCR or serology, that can be limited by the phase of infection or cross-reactivity, the development of metabolic profiling-based diagnostics could enhance faster detection and differential diagnosis for arboviral infection. For example, our study’s ROC curve for the metabolite set identified via PLS-DA demonstrated 96% sensitivity. This finding suggests that metabolomics could assist in dengue diagnosis by detecting altered levels of multiple molecules, instead of relying on the detection of a single compound.

The untargeted metabolomic approach offers valuable insights into the underlying metabolic mechanisms associated with dengue fever. Comparing metabolic profiles between dengue patients and controls revealed a tendency toward separation consistent with clinical symptoms. Thus, metabolomics may serve as a complementary tool in public heath, by the use od biomarker panels for screening, prevention of severe forms of dengue and ultimately assisting on outbreak management in countries where arboviral infections are endemic [54]. Additionally, the metabolites identified in this study warrant further investigation as potential therapeutic targets for dengue.

## Supporting information

S1 Fig Principal Component Analysis score plot including quality control samples. Dengue (QCD) and control (QCC) groups quality control samples showed reliability of data by clustering closely to controls and dengue samples.
(DOCX)

S2 Fig Power analysis calculation for the number of samples used in the study (A) and to achieve a predicted power of 0.8 (B). (DOCX)

S1 Table Extracted ion chromatogram building parameters. (DOCX)

S2 Table Top 15 metabolites ranked by the permutation test from the PLS-DA cross validation. The classification using 5 components was selected as the best classifier for the Variable Importance in Projection.
(DOCX)

S3 Table Metabolite attribution complete list. Metabolites were identified by LipidMaps.
(DOCX)
