# Correction: Dengue virus infection in children: Serum lipidomics profiling for biomarker discovery

**DOI:** 10.1371/journal.pntd.0013862

**Published:** 2025-12-30

**Authors:** Ricardo E. Correa Fierro, Noroska Gabriela Salazar Mogollón, Washington B. Cárdenas, Evencio Joel Medina-Villamizar, Jefferson Pastuña-Fasso, Melanie Ochoa-Ocampo, Giovanna Morán-Marcillo, Mary Ernestina Regato Arrata, Mildred Zambrano, Joyce Andrade, Juan Chang, Saurabh Mehta, Fernanda Bertuccez Cordeiro

The second author’s last name is incorrect. The correct name is: Noroska Gabriela Salazar Mogollón. The correct citation is: Fierro REC, Mogollón NGS, Cárdenas WB, Medina-Villamizar EJ, Pastuña-Fasso J, Ochoa-Ocampo M, et al. (2025) Dengue virus infection in children: Serum lipidomics profiling for biomarker discovery. PLoS Negl Trop Dis 19 (11): e0013691. https://doi.org/10.1371/journal.pntd.0013691.
