# Supplementary material for: Dengue virus infection in children: Serum lipidomics profiling for biomarker discovery
Source: PLoS Negl Trop Dis. 2025 Nov 24;19(11):e0013691. doi: 10.1371/journal.pntd.0013691 (PMC12643310; doi:10.1371/journal.pntd.0013691)
Supplement: S1 Fig — Dengue (QCD) and control (QCC) groups quality control samples showed reliability of data by clustering closely to controls and dengue samples. (DOCX) [file pntd.0013691.s001.docx]

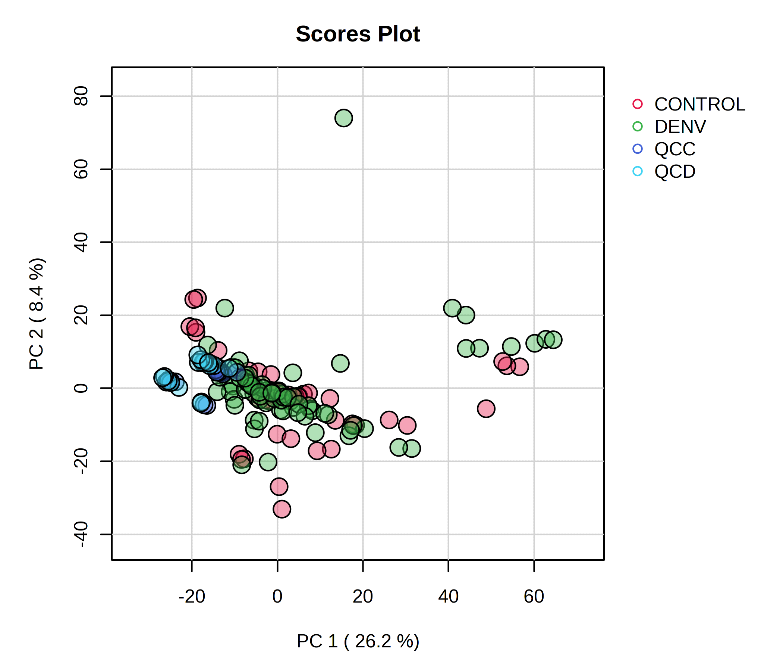


**S1 Fig**: Principal Component Analysis score plot including quality control samples. Dengue (QCD) and control (QCC) groups quality control samples showed reliability of data by clustering closely to controls and dengue samples.
