# Supplementary material for: Dengue virus infection in children: Serum lipidomics profiling for biomarker discovery
Source: PLoS Negl Trop Dis. 2025 Nov 24;19(11):e0013691. doi: 10.1371/journal.pntd.0013691 (PMC12643310; doi:10.1371/journal.pntd.0013691)
Supplement: S2 Fig — (DOCX) [file pntd.0013691.s002.docx]

**S2 Fig**: Power analysis calculation for the number of samples used in the study (A) and to achieve a predicted power of 0.8 (B).
