# Supplementary material for: Dengue virus infection in children: Serum lipidomics profiling for biomarker discovery
Source: PLoS Negl Trop Dis. 2025 Nov 24;19(11):e0013691. doi: 10.1371/journal.pntd.0013691 (PMC12643310; doi:10.1371/journal.pntd.0013691)
Supplement: S1 Table — (DOCX) [file pntd.0013691.s003.docx]

**S1 Table**: Extracted ion chromatogram building parameters

| **Raw Data Method** | Mass detection | **MS Level** | 1 |
| --- | --- | --- | --- |
| **Noise level** | 5.0E1 | **Data type** | Centroid |
| **Feature detection** | ADAP Chromatogram Builder |  |  |
| **Min group size in # of scans** | 4 |  |  |
| **Group intensity threshold** | 1.2E3 |  |  |
| **Min highest intensity** | 6.0E3 |  |  |
| **Scan to scan accuracy** | 0.0050 *m/z* |  |  |
| **Local minimum feature resolver** | | **Dimension** | Retention time |
| **Chromatographic threshold** | 95% |  |  |
| **Minimum absolute height** | 7.0E3 | **Min # of data points** | 4 |
| **Min ratio of peak top/edge** | 1.70 |  |  |
| **Peak smoothing method** | Loess smoothing |  |  |
| **Isotope filter** | 13C isotope grouper | **RT tolerance** | 0.03min |
| **Representative isotope** | Most intense | **m/z tolerance** | 0.002 *m/z* |
| **Alignment method** | Join aligner |  |  |
| **Weight for RT** | 1 | **RT tolerance** | 0.1min |
| **Weight for *m/z*** | 3 | ***m/z* tolerance** | 0.005 *m/z* |
| **Gap filling method** | Peak finder (multithreaded) | |  |
| **Intensity tolerance** | 50% | **RT tolerance** | 0.1min |
| **Min data points** | 2 | ***m/z* tolerance** | 0.002 *m/z* |
