# Supplementary material for: Dengue virus infection in children: Serum lipidomics profiling for biomarker discovery
Source: PLoS Negl Trop Dis. 2025 Nov 24;19(11):e0013691. doi: 10.1371/journal.pntd.0013691 (PMC12643310; doi:10.1371/journal.pntd.0013691)
Supplement: S2 Table — The classification using 5 components was selected as the best classifier for the Variable Importance in Projection. (DOCX) [file pntd.0013691.s004.docx]

**S2 Table**: Top 15 metabolites ranked by the permutation test from the PLS-DA cross validation. The classification using 5 components was selected as the best classifier for the Variable Importance in Projection.

| Mass | VIP (Comp. 5) | CONTROL | DENGUE | AUC |
| --- | --- | --- | --- | --- |
| 246.2655 | 2.9859 | **↓** | **↑** | 0.79526 |
| 349.26489 | 2.6202 | **↑** | **↓** | 0.69244 |
| 305.22946 | 2.5919 | **↑** | **↓** | 0.69689 |
| 512.55011 | 2.52 | **↑** | **↓** | 0.6997 |
| 758.70825 | 2.5141 | **↑** | **↓** | 0.75941 |
| 305.17508 | 2.4633 | **↑** | **↓** | 0.69037 |
| 393.43518 | 2.2934 | **↑** | **↓** | 0.67319 |
| 349.20367 | 2.2406 | **↑** | **↓** | 0.68385 |
| 128.95227 | 2.1255 | **↓** | **↑** | 0.68978 |
| 780.08936 | 2.0927 | **↑** | **↓** | 0.65274 |
| 274.60077 | 2.0751 | **↓** | **↑** | 0.7443 |
| 139.98825 | 2.0441 | **↑** | **↓** | 0.62133 |
| 437.47452 | 2.0028 | **↑** | **↓** | 0.59022 |
| 484.47333 | 1.9987 | **↑** | **↓** | 0.6403 |
| 246.54501 | 1.9541 | **↑** | **↓** | 0.62281 |
| 139.99913 | 1.9317 | **↓** | **↑** | 0.68741 |
