# Supplementary material for: Dengue virus infection in children: Serum lipidomics profiling for biomarker discovery
Source: PLoS Negl Trop Dis. 2025 Nov 24;19(11):e0013691. doi: 10.1371/journal.pntd.0013691 (PMC12643310; doi:10.1371/journal.pntd.0013691)
Supplement: S3 Table — Metabolites were identified by LipidMaps. (DOCX) [file pntd.0013691.s005.docx]

**S3 Table**: Metabolite attribution complete list. Metabolites were identified by LipidMaps.

| **Input Mass** | **Matched Mass** | **Delta** | **Name** | **Formula** | **Ion** |
| --- | --- | --- | --- | --- | --- |
| 246.2655 | 246.2427 | 0.0228 | SPB 14:0;O2 | C14H31NO2 | [M+H]+ |
| 246.2655 | 246.2427 | 0.0228 | FA 14:0 | C14H28O2 | [M+NH4]+ |
| 349.26489 | 349.2585 | 0.0064 | FA 18:0;O4 | C18H36O6 | [M+H]+ |
| 349.26489 | 349.2737 | 0.0088 | FA 22:4;O | C22H36O3 | [M+H]+ |
| 349.26489 | 349.2737 | 0.0088 | ST 22:1;O3 | C22H36O3 | [M+H]+ |
| 349.26489 | 349.2373 | 0.0276 | MG 18:5 | C21H32O4 | [M+H]+ |
| 349.26489 | 349.2737 | 0.0088 | FA 22:3;O2 | C22H38O4 | [M+H-H2O]+ |
| 349.26489 | 349.2737 | 0.0088 | ST 22:0;O4 | C22H38O4 | [M+H-H2O]+ |
| 349.26489 | 349.289 | 0.0241 | ST 26:4;O | C26H38O | [M+H-H2O]+ |
| 349.26489 | 349.2373 | 0.0276 | MG 18:4;O | C21H34O5 | [M+H-H2O]+ |
| 349.26489 | 349.3101 | 0.0452 | MG O-20:3 | C23H42O3 | [M+H-H2O]+ |
| 349.26489 | 349.2162 | 0.0487 | ST 24:6;O3 | C24H30O3 | [M+H-H2O]+ |
| 349.26489 | 349.2713 | 0.0064 | FA 20:1;O | C20H38O3Na | [M+Na]+ |
| 349.26489 | 349.2349 | 0.03 | MG 16:2 | C19H34O4Na | [M+Na]+ |
| 349.26489 | 349.2349 | 0.03 | MG O-16:3;O | C19H34O4Na | [M+Na]+ |
| 349.26489 | 349.2697 | 0.0048 | CAR 10:0;O | C17H33NO5 | [M+NH4]+ |
| 349.26489 | 349.2333 | 0.0316 | NAE 14:2;O4 | C16H29NO6 | [M+NH4]+ |
| 349.26489 | 349.3061 | 0.0412 | NAE 16:0;O2 | C18H37NO4 | [M+NH4]+ |
| 349.26489 | 349.3061 | 0.0412 | SPB 18:1;O4 | C18H37NO4 | [M+NH4]+ |
| 349.26489 | 349.2156 | 0.0493 | NAT 14:2 | C16H29NO4S | [M+NH4]+ |
| 349.26489 | 349.2503 | 0.0146 | FA 20:1 | C20H38O2K | [M+K]+ |
| 349.26489 | 349.2676 | 0.0027 | SM 34:4;O2 | C39H73N2O6P | [M+2H]2+ |
| 349.26489 | 349.26 | 0.0049 | SM 30:0;O5 | C35H73N2O9P | [M+2H]2+ |
| 349.26489 | 349.2555 | 0.0094 | DG 40:8;O2 | C43H68O7 | [M+2H]2+ |
| 349.26489 | 349.2555 | 0.0094 | TG 40:7;O | C43H68O7 | [M+2H]2+ |
| 349.26489 | 349.2555 | 0.0094 | TG O-40:8;O2 | C43H68O7 | [M+2H]2+ |
| 349.26489 | 349.2543 | 0.0106 | LPG 30:0;O | C36H73O10P | [M+2H]2+ |
| 349.26489 | 349.2543 | 0.0106 | PG O-30:0;O | C36H73O10P | [M+2H]2+ |
| 349.26489 | 349.2494 | 0.0155 | CerPE 36:5;O3 | C38H69N2O7P | [M+2H]2+ |
| 349.26489 | 349.2479 | 0.017 | MGDG 30:3 | C39H68O10 | [M+2H]2+ |
| 349.26489 | 349.2843 | 0.0194 | TG 38:1;O2 | C41H76O8 | [M+2H]2+ |
| 349.26489 | 349.2843 | 0.0194 | TG O-38:2;O3 | C41H76O8 | [M+2H]2+ |
| 349.26489 | 349.2438 | 0.0211 | PA 36:4 | C39H69O8P | [M+2H]2+ |
| 349.26489 | 349.2438 | 0.0211 | PA O-36:5;O | C39H69O8P | [M+2H]2+ |
| 349.26489 | 349.2438 | 0.0211 | PEth 34:4 | C39H69O8P | [M+2H]2+ |
| 349.26489 | 349.2418 | 0.0231 | CerPE 32:1;O6 | C34H69N2O10P | [M+2H]2+ |
| 349.26489 | 349.2607 | 0.0041 | CE 16:2;O2 | C43H72O4Na2 | [M+2Na]2+ |
| 349.26489 | 349.2607 | 0.0041 | DG O-40:7 | C43H72O4Na2 | [M+2Na]2+ |
| 349.26489 | 349.2531 | 0.0118 | DG 36:2;O2 | C39H72O7Na2 | [M+2Na]2+ |
| 349.26489 | 349.2531 | 0.0118 | TG 36:1;O | C39H72O7Na2 | [M+2Na]2+ |
| 349.26489 | 349.2531 | 0.0118 | TG O-36:2;O2 | C39H72O7Na2 | [M+2Na]2+ |
| 349.26489 | 349.2895 | 0.0246 | DG 38:0 | C41H80O5Na2 | [M+2Na]2+ |
| 349.26489 | 349.2895 | 0.0246 | DG O-38:1;O | C41H80O5Na2 | [M+2Na]2+ |
| 349.26489 | 349.2895 | 0.0246 | TG O-38:0 | C41H80O5Na2 | [M+2Na]2+ |
| 349.26489 | 349.2325 | 0.0324 | MG O-14:0;O | C17H36O4Na2 | [M+2Na-H]+ |
| 349.26489 | 349.2561 | 0.0088 | MG 16:2;O | C19H34O5Li | [M+Li]+ |
| 349.26489 | 349.2925 | 0.0276 | FA 20:1;O2 | C20H38O4Li | [M+Li]+ |
| 349.26489 | 349.2349 | 0.0299 | ST 22:4;O3 | C22H30O3Li | [M+Li]+ |
| 349.26489 | 349.3077 | 0.0428 | ST 24:2;O | C24H38OLi | [M+Li]+ |
| 349.26489 | 349.2197 | 0.0452 | FA 18:3;O4 | C18H30O6Li | [M+Li]+ |
| 349.26489 | 349.2197 | 0.0452 | ST 18:0;O6 | C18H30O6Li | [M+Li]+ |
| 305.22946 | 305.2322 | 0.0028 | FA 16:0;O3 | C16H32O5 | [M+H]+ |
| 305.22946 | 305.2475 | 0.018 | FA 20:4 | C20H32O2 | [M+H]+ |
| 305.22946 | 305.2475 | 0.018 | ST 20:1;O2 | C20H32O2 | [M+H]+ |
| 305.22946 | 305.2686 | 0.0392 | MG O-14:0;O | C17H36O4 | [M+H]+ |
| 305.22946 | 305.2475 | 0.018 | FA 20:3;O | C20H34O3 | [M+H-H2O]+ |
| 305.22946 | 305.2475 | 0.018 | ST 20:0;O3 | C20H34O3 | [M+H-H2O]+ |
| 305.22946 | 305.2111 | 0.0183 | MG 16:4 | C19H30O4 | [M+H-H2O]+ |
| 305.22946 | 305.19 | 0.0395 | ST 22:6;O2 | C22H26O2 | [M+H-H2O]+ |
| 305.22946 | 305.2451 | 0.0156 | FA 18:1 | C18H34O2Na | [M+Na]+ |
| 305.22946 | 305.2087 | 0.0208 | MG O-14:3 | C17H30O3Na | [M+Na]+ |
| 305.22946 | 305.1876 | 0.0419 | ST 20:4;O | C20H26ONa | [M+Na]+ |
| 305.22946 | 305.2071 | 0.0224 | NAE 12:2;O3 | C14H25NO5 | [M+NH4]+ |
| 305.22946 | 305.2293 | 0.0002 | DG 36:8 | C39H60O5 | [M+2H]2+ |
| 305.22946 | 305.2293 | 0.0002 | DG O-36:9;O | C39H60O5 | [M+2H]2+ |
| 305.22946 | 305.2293 | 0.0002 | TG O-36:8 | C39H60O5 | [M+2H]2+ |
| 305.22946 | 305.2281 | 0.0013 | LPG O-26:1 | C32H65O8P | [M+2H]2+ |
| 305.22946 | 305.2217 | 0.0078 | TG 32:3;O2 | C35H60O8 | [M+2H]2+ |
| 305.22946 | 305.2217 | 0.0078 | TG O-32:4;O3 | C35H60O8 | [M+2H]2+ |
| 305.22946 | 305.2399 | 0.0104 | ST 30:0;O2;Hex | C36H64O7 | [M+2H]2+ |
| 305.22946 | 305.2176 | 0.0119 | LPA O-32:6 | C35H61O6P | [M+2H]2+ |
| 305.22946 | 305.2155 | 0.0139 | CerPE 28:1;O4 | C30H61N2O8P | [M+2H]2+ |
| 305.22946 | 305.2463 | 0.0169 | LPA O-30:0;O | C33H69O7P | [M+2H]2+ |
| 305.22946 | 305.2099 | 0.0195 | PA 28:0;O | C31H61O9P | [M+2H]2+ |
| 305.22946 | 305.2099 | 0.0195 | PEth 26:0;O | C31H61O9P | [M+2H]2+ |
| 305.22946 | 305.205 | 0.0245 | SM 28:6;O2 | C33H57N2O6P | [M+2H]2+ |
| 305.22946 | 305.2269 | 0.0026 | DG 32:2 | C35H64O5Na2 | [M+2Na]2+ |
| 305.22946 | 305.2269 | 0.0026 | DG O-32:3;O | C35H64O5Na2 | [M+2Na]2+ |
| 305.22946 | 305.2269 | 0.0026 | MG 32:3;O | C35H64O5Na2 | [M+2Na]2+ |
| 305.22946 | 305.2269 | 0.0026 | TG O-32:2 | C35H64O5Na2 | [M+2Na]2+ |
| 305.22946 | 305.2345 | 0.0051 | CE 12:2 | C39H64O2Na2 | [M+2Na]2+ |
| 305.22946 | 305.2152 | 0.0143 | LPA O-28:0 | C31H65O6PNa2 | [M+2Na]2+ |
| 305.22946 | 305.2087 | 0.0208 | FA 34:4;O4 | C34H60O6Na2 | [M+2Na]2+ |
| 305.22946 | 305.2087 | 0.0208 | ST 28:0;O;Hex | C34H60O6Na2 | [M+2Na]2+ |
| 305.22946 | 305.2063 | 0.0232 | MG O-12:0 | C15H32O3Na2 | [M+2Na-H]+ |
| 305.22946 | 305.1852 | 0.0443 | ST 18:1;O | C18H28ONa2 | [M+2Na-H]+ |
| 305.22946 | 305.2299 | 0.0004 | MG 14:2 | C17H30O4Li | [M+Li]+ |
| 305.22946 | 305.2299 | 0.0004 | MG O-14:3;O | C17H30O4Li | [M+Li]+ |
| 305.22946 | 305.2087 | 0.0207 | ST 20:4;O2 | C20H26O2Li | [M+Li]+ |
| 305.22946 | 305.1935 | 0.036 | FA 16:3;O3 | C16H26O5Li | [M+Li]+ |
| 305.22946 | 305.2662 | 0.0368 | FA 18:1;O | C18H34O3Li | [M+Li]+ |
| 512.55011 | 512.5037 | 0.0464 | Cer 32:0;O2 | C32H65NO3 | [M+H]+ |
| 512.55011 | 512.5037 | 0.0464 | NAE 30:0;O | C32H65NO3 | [M+H]+ |
| 512.55011 | 512.5037 | 0.0464 | FA 32:1;O | C32H62O3 | [M+NH4]+ |
| 758.70825 | 758.7385 | 0.0302 | Cer 50:3;O2 | C50H95NO3 | [M+H]+ |
| 758.70825 | 758.6657 | 0.0426 | ACer 48:4;O3 | C48H87NO5 | [M+H]+ |
| 758.70825 | 758.6657 | 0.0426 | Cer 48:5;O4 | C48H87NO5 | [M+H]+ |
| 758.70825 | 758.7385 | 0.0302 | ACer 50:1;O2 | C50H97NO4 | [M+H-H2O]+ |
| 758.70825 | 758.7385 | 0.0302 | Cer 50:2;O3 | C50H97NO4 | [M+H-H2O]+ |
| 758.70825 | 758.6657 | 0.0426 | ACer 48:3;O4 | C48H89NO6 | [M+H-H2O]+ |
| 758.70825 | 758.6657 | 0.0426 | Cer 48:4;O5 | C48H89NO6 | [M+H-H2O]+ |
| 758.70825 | 758.7361 | 0.0278 | Cer 48:0;O2 | C48H97NO3Na | [M+Na]+ |
| 758.70825 | 758.6785 | 0.0297 | Cer 50:6;O | C50H89NO2Na | [M+Na]+ |
| 758.70825 | 758.6633 | 0.045 | ACer 46:1;O3 | C46H89NO5Na | [M+Na]+ |
| 758.70825 | 758.6633 | 0.045 | Cer 46:2;O4 | C46H89NO5Na | [M+Na]+ |
| 758.70825 | 758.7021 | 0.0062 | CE 22:0;O2 | C49H88O4 | [M+NH4]+ |
| 758.70825 | 758.7021 | 0.0062 | DG O-46:5 | C49H88O4 | [M+NH4]+ |
| 758.70825 | 758.6868 | 0.0214 | DG 42:0;O2 | C45H88O7 | [M+NH4]+ |
| 758.70825 | 758.6868 | 0.0214 | TG O-42:0;O2 | C45H88O7 | [M+NH4]+ |
| 758.70825 | 758.7151 | 0.0068 | Cer 48:0;O | C48H97NO2K | [M+K]+ |
| 758.70825 | 758.7041 | 0.0042 | TG 100:10 | C103H182O6 | [M+2H]2+ |
| 758.70825 | 758.7041 | 0.0042 | TG O-100:11;O | C103H182O6 | [M+2H]2+ |
| 758.70825 | 758.6965 | 0.0118 | TG 96:6;O3 | C99H182O9 | [M+2H]2+ |
| 758.70825 | 758.7328 | 0.0246 | TG 98:4;O | C101H190O7 | [M+2H]2+ |
| 758.70825 | 758.7328 | 0.0246 | TG O-98:5;O2 | C101H190O7 | [M+2H]2+ |
| 758.70825 | 758.7017 | 0.0066 | TG 96:4 | C99H186O6Na2 | [M+2Na]2+ |
| 758.70825 | 758.7017 | 0.0066 | TG O-96:5;O | C99H186O6Na2 | [M+2Na]2+ |
| 758.70825 | 758.6941 | 0.0142 | TG 92:0;O3 | C95H186O9Na2 | [M+2Na]2+ |
| 758.70825 | 758.6761 | 0.0321 | Cer 48:3;O | C48H91NO2Na2 | [M+2Na-H]+ |
| 758.70825 | 758.6997 | 0.0086 | Cer 50:6;O2 | C50H89NO3Li | [M+Li]+ |
| 758.70825 | 758.6844 | 0.0238 | ACer 46:1;O4 | C46H89NO6Li | [M+Li]+ |
| 758.70825 | 758.6844 | 0.0238 | Cer 46:2;O5 | C46H89NO6Li | [M+Li]+ |
| 758.70825 | 758.7572 | 0.049 | Cer 48:0;O3 | C48H97NO4Li | [M+Li]+ |
| 305.17508 | 305.1747 | 0.0004 | ST 18:3;O4 | C18H24O4 | [M+H]+ |
| 305.17508 | 305.1747 | 0.0004 | FA 18:5;O3 | C18H26O5 | [M+H-H2O]+ |
| 305.17508 | 305.1747 | 0.0004 | ST 18:2;O5 | C18H26O5 | [M+H-H2O]+ |
| 305.17508 | 305.19 | 0.0149 | ST 22:6;O2 | C22H26O2 | [M+H-H2O]+ |
| 305.17508 | 305.2111 | 0.036 | MG 16:4 | C19H30O4 | [M+H-H2O]+ |
| 305.17508 | 305.1723 | 0.0028 | FA 16:3;O2 | C16H26O4Na | [M+Na]+ |
| 305.17508 | 305.1876 | 0.0125 | ST 20:4;O | C20H26ONa | [M+Na]+ |
| 305.17508 | 305.2087 | 0.0336 | MG O-14:3 | C17H30O3Na | [M+Na]+ |
| 305.17508 | 305.2071 | 0.032 | NAE 12:2;O3 | C14H25NO5 | [M+NH4]+ |
| 305.17508 | 305.1513 | 0.0237 | FA 16:3;O | C16H26O3K | [M+K]+ |
| 305.17508 | 305.1747 | 0.0004 | ST 30:7;O2;GlcA | C36H48O8 | [M+2H]2+ |
| 305.17508 | 305.1735 | 0.0015 | LPI O-20:3 | C29H53O11P | [M+2H]2+ |
| 305.17508 | 305.1812 | 0.0061 | LPA 30:7;O | C33H53O8P | [M+2H]2+ |
| 305.17508 | 305.1812 | 0.0061 | PA 30:6 | C33H53O8P | [M+2H]2+ |
| 305.17508 | 305.1812 | 0.0061 | PA O-30:7;O | C33H53O8P | [M+2H]2+ |
| 305.17508 | 305.1812 | 0.0061 | PEth 28:6 | C33H53O8P | [M+2H]2+ |
| 305.17508 | 305.1671 | 0.008 | ST 26:3;O5;GlcA | C32H48O11 | [M+2H]2+ |
| 305.17508 | 305.1671 | 0.008 | ST 26:4;O6;Hex | C32H48O11 | [M+2H]2+ |
| 305.17508 | 305.1853 | 0.0102 | MGDG 24:5 | C33H52O10 | [M+2H]2+ |
| 305.17508 | 305.1917 | 0.0167 | BMP 24:1 | C30H57O10P | [M+2H]2+ |
| 305.17508 | 305.1917 | 0.0167 | LPG 24:2;O | C30H57O10P | [M+2H]2+ |
| 305.17508 | 305.1917 | 0.0167 | PG 24:1 | C30H57O10P | [M+2H]2+ |
| 305.17508 | 305.1917 | 0.0167 | PG O-24:2;O | C30H57O10P | [M+2H]2+ |
| 305.17508 | 305.1929 | 0.0178 | TG 34:9;O | C37H52O7 | [M+2H]2+ |
| 305.17508 | 305.1723 | 0.0028 | ST 26:1;O2;GlcA | C32H52O8Na2 | [M+2Na]2+ |
| 305.17508 | 305.1723 | 0.0028 | ST 26:2;O3;Hex | C32H52O8Na2 | [M+2Na]2+ |
| 305.17508 | 305.1788 | 0.0037 | LPA 26:1;O | C29H57O8PNa2 | [M+2Na]2+ |
| 305.17508 | 305.1788 | 0.0037 | PA 26:0 | C29H57O8PNa2 | [M+2Na]2+ |
| 305.17508 | 305.1788 | 0.0037 | PA O-26:1;O | C29H57O8PNa2 | [M+2Na]2+ |
| 305.17508 | 305.1788 | 0.0037 | PEth 24:0 | C29H57O8PNa2 | [M+2Na]2+ |
| 305.17508 | 305.1618 | 0.0133 | TG 32:9 | C35H48O6Na2 | [M+2Na]2+ |
| 305.17508 | 305.1606 | 0.0145 | LPG 22:2 | C28H53O9PNa2 | [M+2Na]2+ |
| 305.17508 | 305.1606 | 0.0145 | LPG O-22:3;O | C28H53O9PNa2 | [M+2Na]2+ |
| 305.17508 | 305.1606 | 0.0145 | PG O-22:2 | C28H53O9PNa2 | [M+2Na]2+ |
| 305.17508 | 305.1905 | 0.0154 | DG 30:4;O2 | C33H56O7Na2 | [M+2Na]2+ |
| 305.17508 | 305.1905 | 0.0154 | TG 30:3;O | C33H56O7Na2 | [M+2Na]2+ |
| 305.17508 | 305.1905 | 0.0154 | TG O-30:4;O2 | C33H56O7Na2 | [M+2Na]2+ |
| 305.17508 | 305.1981 | 0.0231 | DG O-34:9 | C37H56O4Na2 | [M+2Na]2+ |
| 305.17508 | 305.1699 | 0.0052 | FA 14:0;O2 | C14H28O4Na2 | [M+2Na-H]+ |
| 305.17508 | 305.1852 | 0.0101 | ST 18:1;O | C18H28ONa2 | [M+2Na-H]+ |
| 305.17508 | 305.2063 | 0.0312 | MG O-12:0 | C15H32O3Na2 | [M+2Na-H]+ |
| 305.17508 | 305.1335 | 0.0415 | MG 10:1;O | C13H24O5Na2 | [M+2Na-H]+ |
| 305.17508 | 305.1935 | 0.0184 | FA 16:3;O3 | C16H26O5Li | [M+Li]+ |
| 305.17508 | 305.2087 | 0.0337 | ST 20:4;O2 | C20H26O2Li | [M+Li]+ |
| 349.20367 | 349.2009 | 0.0027 | ST 20:3;O5 | C20H28O5 | [M+H]+ |
| 349.20367 | 349.2373 | 0.0337 | MG 18:5 | C21H32O4 | [M+H]+ |
| 349.20367 | 349.2009 | 0.0027 | FA 20:5;O4 | C20H30O6 | [M+H-H2O]+ |
| 349.20367 | 349.2009 | 0.0027 | ST 20:2;O6 | C20H30O6 | [M+H-H2O]+ |
| 349.20367 | 349.2138 | 0.0102 | LPA O-14:1 | C17H35O6P | [M+H-H2O]+ |
| 349.20367 | 349.2162 | 0.0125 | ST 24:6;O3 | C24H30O3 | [M+H-H2O]+ |
| 349.20367 | 349.1832 | 0.0205 | ST 20:2;O;S | C20H30O4S | [M+H-H2O]+ |
| 349.20367 | 349.2373 | 0.0337 | MG 18:4;O | C21H34O5 | [M+H-H2O]+ |
| 349.20367 | 349.1985 | 0.0051 | FA 18:3;O3 | C18H30O5Na | [M+Na]+ |
| 349.20367 | 349.1985 | 0.0051 | ST 18:0;O5 | C18H30O5Na | [M+Na]+ |
| 349.20367 | 349.2138 | 0.0101 | ST 22:4;O2 | C22H30O2Na | [M+Na]+ |
| 349.20367 | 349.2349 | 0.0313 | MG 16:2 | C19H34O4Na | [M+Na]+ |
| 349.20367 | 349.2349 | 0.0313 | MG O-16:3;O | C19H34O4Na | [M+Na]+ |
| 349.20367 | 349.2156 | 0.0119 | NAT 14:2 | C16H29NO4S | [M+NH4]+ |
| 349.20367 | 349.2333 | 0.0296 | NAE 14:2;O4 | C16H29NO6 | [M+NH4]+ |
| 349.20367 | 349.2139 | 0.0103 | MG O-16:3 | C19H34O3K | [M+K]+ |
| 349.20367 | 349.1928 | 0.0109 | ST 22:4;O | C22H30OK | [M+K]+ |
| 349.20367 | 349.1776 | 0.0261 | FA 18:3;O2 | C18H30O4K | [M+K]+ |
| 349.20367 | 349.1776 | 0.0261 | ST 18:0;O4 | C18H30O4K | [M+K]+ |
| 349.20367 | 349.2503 | 0.0467 | FA 20:1 | C20H38O2K | [M+K]+ |
| 349.20367 | 349.1998 | 0.0039 | LPI 24:2;O | C33H61O13P | [M+2H]2+ |
| 349.20367 | 349.1998 | 0.0039 | PI 24:1 | C33H61O13P | [M+2H]2+ |
| 349.20367 | 349.1998 | 0.0039 | PI O-24:2;O | C33H61O13P | [M+2H]2+ |
| 349.20367 | 349.1933 | 0.0104 | ST 30:3;O7;GlcA | C36H56O13 | [M+2H]2+ |
| 349.20367 | 349.1933 | 0.0104 | ST 30:4;O8;Hex | C36H56O13 | [M+2H]2+ |
| 349.20367 | 349.1892 | 0.0145 | BMP 30:7;O | C36H57O11P | [M+2H]2+ |
| 349.20367 | 349.1892 | 0.0145 | PG 30:7;O | C36H57O11P | [M+2H]2+ |
| 349.20367 | 349.2191 | 0.0155 | TG 38:9;O3 | C41H60O9 | [M+2H]2+ |
| 349.20367 | 349.2256 | 0.0219 | LPG 32:6 | C38H65O9P | [M+2H]2+ |
| 349.20367 | 349.2256 | 0.0219 | LPG O-32:7;O | C38H65O9P | [M+2H]2+ |
| 349.20367 | 349.2256 | 0.0219 | PG O-32:6 | C38H65O9P | [M+2H]2+ |
| 349.20367 | 349.2 | 0.0036 | SM 30:6;O3 | C35H61N2O7PNa2 | [M+2Na]2+ |
| 349.20367 | 349.1985 | 0.0051 | ST 30:1;O4;GlcA | C36H60O10Na2 | [M+2Na]2+ |
| 349.20367 | 349.1985 | 0.0051 | ST 30:2;O5;Hex | C36H60O10Na2 | [M+2Na]2+ |
| 349.20367 | 349.2106 | 0.0069 | CerPE 30:1;O5 | C32H65N2O9PNa2 | [M+2Na]2+ |
| 349.20367 | 349.2126 | 0.0089 | LPA 34:5 | C37H65O7PNa2 | [M+2Na]2+ |
| 349.20367 | 349.2126 | 0.0089 | LPA O-34:6;O | C37H65O7PNa2 | [M+2Na]2+ |
| 349.20367 | 349.2126 | 0.0089 | PA O-34:5 | C37H65O7PNa2 | [M+2Na]2+ |
| 349.20367 | 349.1944 | 0.0093 | LPG O-30:7 | C36H61O8PNa2 | [M+2Na]2+ |
| 349.20367 | 349.2167 | 0.0131 | TG 34:3;O3 | C37H64O9Na2 | [M+2Na]2+ |
| 349.20367 | 349.2182 | 0.0146 | CerPE 34:5;O2 | C36H65N2O6PNa2 | [M+2Na]2+ |
| 349.20367 | 349.188 | 0.0157 | TG 36:9;O2 | C39H56O8Na2 | [M+2Na]2+ |
| 349.20367 | 349.188 | 0.0157 | TG O-36:10;O3 | C39H56O8Na2 | [M+2Na]2+ |
| 349.20367 | 349.1868 | 0.0169 | BMP 26:1;O | C32H61O11PNa2 | [M+2Na]2+ |
| 349.20367 | 349.1868 | 0.0169 | PG 26:1;O | C32H61O11PNa2 | [M+2Na]2+ |
| 349.20367 | 349.2232 | 0.0195 | LPG 28:0 | C34H69O9PNa2 | [M+2Na]2+ |
| 349.20367 | 349.2232 | 0.0195 | LPG O-28:1;O | C34H69O9PNa2 | [M+2Na]2+ |
| 349.20367 | 349.2232 | 0.0195 | PG O-28:0 | C34H69O9PNa2 | [M+2Na]2+ |
| 349.20367 | 349.2244 | 0.0207 | CE 14:4;O4 | C41H64O6Na2 | [M+2Na]2+ |
| 349.20367 | 349.2244 | 0.0207 | DG 38:8;O | C41H64O6Na2 | [M+2Na]2+ |
| 349.20367 | 349.2244 | 0.0207 | DG O-38:9;O2 | C41H64O6Na2 | [M+2Na]2+ |
| 349.20367 | 349.2244 | 0.0207 | TG 38:7 | C41H64O6Na2 | [M+2Na]2+ |
| 349.20367 | 349.2244 | 0.0207 | TG O-38:8;O | C41H64O6Na2 | [M+2Na]2+ |
| 349.20367 | 349.1961 | 0.0075 | FA 16:0;O3 | C16H32O5Na2 | [M+2Na-H]+ |
| 349.20367 | 349.2114 | 0.0077 | FA 20:4 | C20H32O2Na2 | [M+2Na-H]+ |
| 349.20367 | 349.2114 | 0.0077 | ST 20:1;O2 | C20H32O2Na2 | [M+2Na-H]+ |
| 349.20367 | 349.2325 | 0.0289 | MG O-14:0;O | C17H36O4Na2 | [M+2Na-H]+ |
| 349.20367 | 349.2019 | 0.0017 | ST 18:0;O;S | C18H30O4SLi | [M+Li]+ |
| 349.20367 | 349.2197 | 0.016 | FA 18:3;O4 | C18H30O6Li | [M+Li]+ |
| 349.20367 | 349.2197 | 0.016 | ST 18:0;O6 | C18H30O6Li | [M+Li]+ |
| 349.20367 | 349.2349 | 0.0313 | ST 22:4;O3 | C22H30O3Li | [M+Li]+ |
| 349.20367 | 349.1598 | 0.0439 | LPA 10:0;O | C13H27O8PLi | [M+Li]+ |
| 274.60077 | 274.6193 | 0.0185 | ST 26:7;O5;Tau | C28H37NO8S | [M+2H]2+ |
| 274.60077 | 274.6034 | 0.0027 | LPT 16:4 | C23H38NO10PNa2 | [M+2Na]2+ |
| 274.60077 | 274.597 | 0.0038 | ST 18:5;O4;HexNAc | C26H33NO9Na2 | [M+2Na]2+ |
| 274.60077 | 274.597 | 0.0038 | ST 24:6;O7;Gly | C26H33NO9Na2 | [M+2Na]2+ |
| 274.60077 | 274.6169 | 0.0161 | NAT 22:4;O4 | C24H41NO8SNa2 | [M+2Na]2+ |
| 274.60077 | 274.6169 | 0.0161 | ST 22:1;O5;Tau | C24H41NO8SNa2 | [M+2Na]2+ |
| 274.60077 | 274.5805 | 0.0203 | ST 20:3;O7;Tau | C22H33NO10SNa2 | [M+2Na]2+ |
| 274.60077 | 274.6216 | 0.0208 | LPC 16:4;O | C24H42NO8PNa2 | [M+2Na]2+ |
| 274.60077 | 274.6216 | 0.0208 | LPS O-18:4 | C24H42NO8PNa2 | [M+2Na]2+ |
| 274.60077 | 274.6245 | 0.0237 | ST 26:5;O2;Tau | C28H41NO5SNa2 | [M+2Na]2+ |
| 437.47452 | 437.4353 | 0.0392 | MG O-26:1 | C29H58O3 | [M+H-H2O]+ |
| 437.47452 | 437.4329 | 0.0416 | ST 30:0;O | C30H54OLi | [M+Li]+ |
| 484.47333 | 484.4724 | 0.0009 | Cer 30:0;O2 | C30H61NO3 | [M+H]+ |
| 484.47333 | 484.4724 | 0.0009 | NAE 28:0;O | C30H61NO3 | [M+H]+ |
| 484.47333 | 484.436 | 0.0373 | CAR 22:0 | C29H57NO4 | [M+H]+ |
| 484.47333 | 484.4724 | 0.0009 | FA 30:1;O | C30H58O3 | [M+NH4]+ |
| 484.47333 | 484.436 | 0.0373 | DG O-26:2 | C29H54O4 | [M+NH4]+ |
| 484.47333 | 484.436 | 0.0373 | MG 26:2 | C29H54O4 | [M+NH4]+ |
| 484.47333 | 484.436 | 0.0373 | MG O-26:3;O | C29H54O4 | [M+NH4]+ |
| 484.47333 | 484.4744 | 0.0011 | DG O-62:4 | C65H122O4 | [M+2H]2+ |
| 484.47333 | 484.4336 | 0.0397 | Cer 30:3;O2 | C30H55NO3Li | [M+Li]+ |
| 484.47333 | 484.4336 | 0.0397 | NAE 28:3;O | C30H55NO3Li | [M+Li]+ |
